# Design and Modification of a High-Resolution Optical Interferometer Accelerometer

**DOI:** 10.3390/s21062070

**Published:** 2021-03-16

**Authors:** Yuan Yao, Debin Pan, Jianbo Wang, Tingting Dong, Jie Guo, Chensheng Wang, Anbing Geng, Weidong Fang, Qianbo Lu

**Affiliations:** 1 Wuhan National Lab for Optoelectronics, Huazhong University of Science and Technology, Wuhan 430074, China; pdb@gdjsxjs.onexmail.com; 2 Wuhan National Lab for Optoelectronics, Huazhong Institute of Electro-Optics, Wuhan 430223, China; jianbo@hust.edu.cn (J.W.); 20170901053@cpu.edu.cn (T.D.); Guoj@hust.edu.cn (J.G.); wcs@gdjsxjs.onexmail.com (C.W.); genganbing@gdjsxjs.onexmail.com (A.G.); 3 State Key Laboratory of Modern Optical Instrumentation, Department of Optical Engineering, Zhejiang University, Hangzhou 310027, China; fangwd@zju.edu.cn; 4 Frontiers Science Center for Flexible Electronics (FSCFE), MIIT Key Laboratory of Flexible Electronics (KLoFE), Shaanxi Key Laboratory of Flexible Electronics (KLoFE), Institute of Flexible Electronics (IFE), Ningbo Institute of Northwestern Polytechnical University, Northwestern Polytechnical University, Xi’an 710072, China; iamqlu@nwpu.edu.cn

**Keywords:** MOEMS accelerometer, interferometry, diffraction gratings, low-*g* accelerometer

## Abstract

The Micro-Opto-Electro-Mechanical Systems (MOEMS) accelerometer is a new type of accelerometer that combines the merits of optical measurement and Micro-Electro-Mechanical Systems (MEMS) to enable high precision, small volume, and anti-electromagnetism disturbance measurement of acceleration, which makes it a promising candidate for inertial navigation and seismic monitoring. This paper proposes a modified micro-grating-based accelerometer and introduces a new design method to characterize the grating interferometer. A MEMS sensor chip with high sensitivity was designed and fabricated, and the processing circuit was modified. The micro-grating interference measurement system was modeled, and the response sensitivity was analyzed. The accelerometer was then built and benchmarked with a commercial seismometer in detail. Compared to the previous prototype in the experiment, the results indicate that the noise floor has an ultra-low self-noise of 15 ng/Hz^1/2^.

## 1. Introduction

Recently, the micro-optical electronic mechanical system (MOEMS) accelerometers have been a public focus because of the significant advantages of optical sensors over their conventional counterparts [1]. MOEMS accelerometers combine optical measurements with the micro-electronic mechanical system (MEMS) technology. MOEMS technology has gained increasing attention in the scientific community due to its wide variety of advantages, such as immunity to electromagnetic interference, electrical insulation, corrosion resistance, remote sensing, high sensitivity, and multiplexing ability [2,3,4]. A wide range of applications exists for of this type of accelerometers, including inertial navigation with high accuracy, vibration sensing of vehicles, seismic sensing, and oil-field applications [5,6].

In recent decades, many schemes using different optical techniques have been introduced, including grating interferometry, Fabry–Perot cavity, fiber Bragg grating, photonic crystal nano-cavity, light force, and evanescent wave (EW) coupling [7,8,9,10,11,12,13,14]. Among these above-mentioned methods, interferometry accelerometer designs using micro-gratings have the potential to achieve a more compact size and a higher sensitivity compared with other types, which is verified by the authors of [15,16].

Interferometers based on diffraction gratings have been widely used in displacement measurement [9]. Since the low thermal expansion coefficient of the grating on a quartz substrate, grating interferometers have the features of high reliability, high sensitivity, and small zero deviation drift. In recent years, researchers have applied grating interferometers to acceleration sensors and obtained good results [17,18,19,20,21,22,23,24]. In order to further improve the performance of the grating accelerometer, the acceleration and displacement sensing element of the elastic coefficient, the mass block, and the cross-axis suppression ratio needs to be further optimized. The displacement sensor system needs to be more precisely modeled, making the sensors work at the best working point. In addition, a low noise interface circuit should be applied, and noise from the detectors and laser, such as 1/f noise, thermal noise, and relative intensity noise (RIN) should be carefully considered.

This paper introduces the theory, simulation, and experimental demonstration of a modified MOEMS accelerometer based on a diffraction grating. This accelerometer can achieve a high sensitivity and resolution in acceleration measurements through the acceleration-sensitive MEMS sensor chip, the compact grating interferometer, and the modified processing circuit. Finite element modeling (FEM) with an optimization of acceleration-displacement (a-d) sensitivity was used for analysis and numerical simulation. Then, the chip and the matching grating were manufactured. The package structure for integrating the vertical cavity surface emitting laser (VCSEL) and multiple photodiode (PD) detectors were designed. In addition, the corresponding optical readout circuit and the final system integrations were completed. In the end, the response sensitivity and self-noise of the system were compared with a commercial seismometer. Compared with the previous design [19], the test results showed that the response sensitivity of the MOEMS accelerometer was about 60V/g and the self-noise decreased from 185.8 ng/√Hz to 15 ng/√Hz. Among the micromachined accelerometers with sub-μg/√Hz noise floor, this type of optical accelerometer has a lower noise floor and is expected to be used in seismic surveys and other fields [2].

## 2. Design

### 2.1. Micromachined Sensing Chip

**Figure 1:**
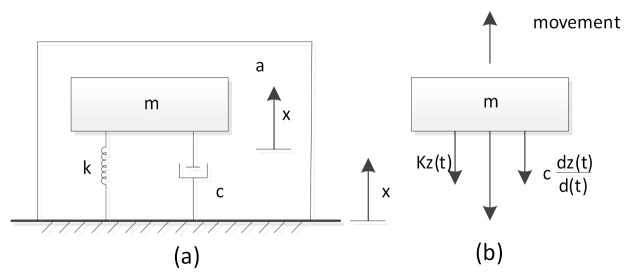
Schematic of the accelerometer. (**a**) Lumped model of the accelerometer. (**b**) The force on the sensitive mass. k is the elastic coefficient of the spring, c is the damping coefficient, x is the relative displacement between the mass and the base, a is the acceleration acting on the model.

The MEMS sensor chip converts the applied acceleration into the displacement of the detection mass, which constitutes a chip with spring support and a mass base. Figure 1a shows a schematic diagram of the sensor chip. As shown in Figure 1b, it can be considered as a second-order mass-spring-damped system, and the four cantilevers can be considered as springs. When the working frequency is much lower than the natural frequency of the structure, the displacement of the mass is proportional to the applied acceleration:

(1) $$d\propto \frac{1}{(2\pi f_{0}{)}^{2}}a=\frac{m}{k}a$$

where *d* is the displacement of the proof mass, *m* is the mass of the proof mass, $f_{0}$ is the natural frequency of the structure, *a* is the acceleration acting on the mode, *x* is the relative displacement between the mass and the base (housing), *c* is the damping coefficient, and *k* is the spring constant. *m/k* is called a-d sensitivity; the higher the a-d sensitivity, the lower *k* and the larger *m*. However, there is a trade-off between a-d sensitivity and bandwidth. In addition, residual stress should be considered thoroughly as it relates to the reliability of the structure.

#### 2.1.1. Sensing Chip Theoretical Design

The total noise of the accelerometer is composed of mechanical thermal noise and electrical noise. The mechanical thermal noise equivalent acceleration ($NEA_{mechanical}$) of the spring-mass accelerometer can be expressed as [25]:

(2) $$NEA_{mechanical}=\sqrt{\frac{8\pi k_{B}Tf_{0}}{mQ}}$$

According to Equations (1) and (2), $NEA_{mechanical}$ can be lowered by improving the weight of the proof mass, reducing the resonant frequency, and improving the quality factor. In general, a massive block is difficult to obtain while reducing the stiffness is a more economical method. According to Equation (3), the stiffness can be reduced by increasing the length of the cantilever beam or reducing the moment of inertia in area. In addition to noise reduction, the working range and bandwidth should be considered as effecting factors. In the actual design, these parameters need to be weighted to obtain the optimal performance.

In practice, accelerations with an arbitrary direction exerted on the accelerometer can result in displacement and tilt of the mass block, thus introducing cross-sensitivities, and a symmetrical design can reduce them [19]. The suspension has symmetrical configurations, where the six cantilever beams are divided into two groups and placed symmetrically on the upper and lower surfaces of the mass block. Therefore, a smaller stiffness on the *Z*-axis and a larger stiffness on the other axes are obtained, and the suppression ratio of the resonant frequency of the cross axis to the fundamental frequency becomes very large.

For a single cantilever beam with one end fixed and the other end guided, if the force *F* is applied on the guided end along the sensitive *Z*-axis, the displacement along that axis can be expressed as [20]

(3) $$\Delta d=\frac{19m_{1}L^{3}}{24EI_{l}}a$$

where $m_{1}$ is the mass of the block, *L* is the length of the mass, $I_{1}$ is the moment of inertia of the cantilever beam, and E is Young’s modulus of elasticity. Equation (3) shows that the displacement change of the sensor mass block is directly proportional to the external acceleration. In addition, the geometric dimensions of the MEMS sensing chip, such as the mass $m_{1}$ of the sensor mass block, the thickness *t,* and the width *w* of the cantilever beam determine the linear scaling factor of the acceleration converted into the displacement. Therefore, a high sensitivity, a low off-axis crosstalk, and a certain bandwidth can be achieved by adjusting the geometric size of the MEMS sensing structure [26].

**Figure 2:**
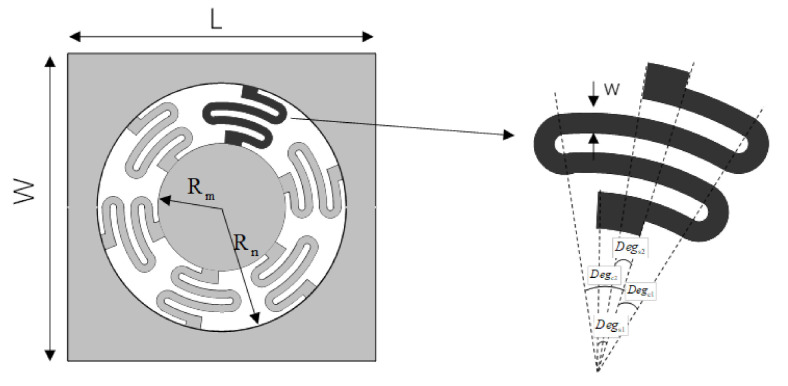
Structure of the spring-mass system.

In order to avoid oscillations and transverse crosstalk in the first-order operation mode and meet the requirements of high sensitivity and low off-axis sensitivity, the MEMS sensor chip also needs to be designed according to the current processing technology conditions. The spring-mass system of the proposed accelerometer is mainly composed of the suspension, the proof mass, and the sensor frame, as shown in Figure 2. An SOI wafer with five layers was selected to make this sensor chip. The mass block is circular while the cantilever beam is uniformly arranged around the mass block. The length, width, and height of the frame (*L × W × T*) are 13 mm × 13 mm × 0.5 mm, respectively. The thickness of cantilever beam *t* is 0.012 mm. Material parameters are as follows: density $\rho =2.33\times 10^{3}{\;\mathrm{kg}/m}^{3}$, Young’s modulus of elasticity $E=1.9\times 10^{11}{\;N/m}^{2}$, and Poisson’s ratio σ=0.25. On account of the thickness of the sensitive mass is much larger than that of the beam, the sensitive mass can be regarded as an ideal rigid body.

The design target of the accelerometer is to have a bandwidth above 10 Hz with a noise floor as low as possible [27]. According to the target specifications, the design parameters of the spring-mass structure were selected and are specified in Table 1.

#### 2.1.2. Finite Element Method (FEM) Analysis

In terms of the analysis, linear static analysis is used to calculate the displacement, stress, and strain of loaded structures with no regard to inertia or damping. In this section, it is assumed that the response of loads and structures changes very rapidly with time. Firstly, the static linear characteristics of the sensing structure are carried out; that is, the displacement of each point on the sensing structure along the direction of the sensing axis is calculated within the range of ±1 *g*. In the static analysis, the ends of the cantilever beam are constrained by six dimensions. When the gravitational acceleration of 1 *g* is applied to the mass block in the direction of the sensitive axis (*Z* axis), the stress distribution exists on the cantilever beam, and the maximum displacement of the mass block obtained by simulation is about 169 µm.

**Figure 3:**
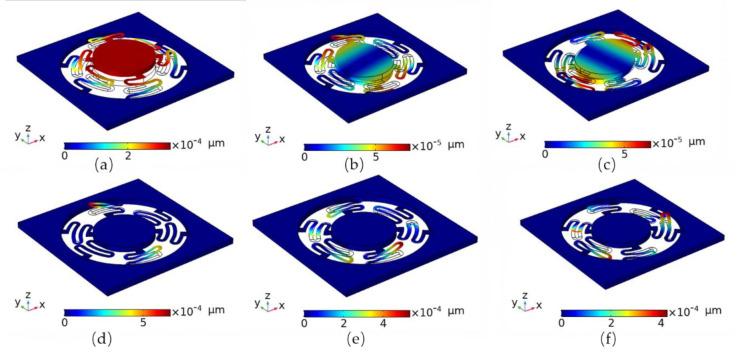
(**a**)~(**f**) The first six order vibration modal analysis results of the Micro-Electro-Mechanical Systems (MEMS) sensor chip. The resonant mode frequencies are approximately 24.8 Hz, 321.5 Hz, 321.6 Hz, 584.9 Hz, 585.4 Hz, 586.1 Hz, respectively.

A modal analysis is used to verify the dynamic capability of the accelerometer [28]. A modal analysis is mainly applied to determine the natural frequency and mode shape of the structure. The displacement and rotational freedom at the fixed end of the cantilever beam is set to zero. The resonance deformation diagram of the first five order resonance modes of the sensing structure was obtained through simulation calculation by COMSOL software, as shown in Figure 3a–f. The stationary solver and eigenvalue solver in the COMSOL Multiphysics software were used. A convergence study has been done, and the maximum cell size governed the convergence. A free tetrahedral mesh method was used in the end, and the error of the mesh we picked with respect to the finest one was 5.2%. The ratio of the insensitive axis frequency to the sensitive axis frequency based on the micro—mechanical sensor structure is shown in Table 2. The cross-sensitivity suppression ratio of the structure designed in this paper is 13.0, which is much higher than 3.1, that of the single-layer cantilever beam structure designed in previous work [19].

From the modal analysis results, the resonant frequency of the first mode and the operating mode are much lower than that of other higher-order modes. Thus, the interference mode of higher-order torsion does not easily occur in the first-order operating mode [29]. In order to obtain higher induction sensitivity, the design of the rotary beam is very thin, and the thickness of the induction mass block is relatively large while the resonant frequency of the first mode structure is not high, which limits the work of the bandwidth micro-mechanical sensing structure to a certain extent.

In addition, through FEM analysis, a 10 *g* acceleration is applied to the direction of the sensitive axis of the structure, and its maximum stress is about 548 MPa, which is far less than the yield strength of silicon material (the yield strength of monocrystalline silicon material is 1.65 GPa). This design can prevent the fracture of the S-shape structure in the release process of the sensitive element. Moreover, curved cantilever beams are adopted in the design to mitigate residual stress concentration at the corner of the flexure. Compared with the previous design, the maximum stress decreases from 32.4 MPa to 23.5 MPa [19].

### 2.2. Optical Displacement Readout

**Figure 4:**
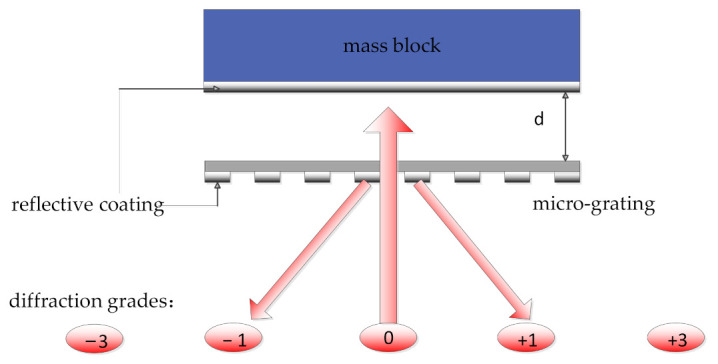
Schematic diagram of the optical displacement readout based on an interferometry cavity. *d* refers the distance between mass block and the micro-grating.

According to Figure 4, the optical displacement readout is based on a grating interferometric readout system, which mainly includes a diffraction grating and a reflection mirror. The output intensity is modulated by the relative displacement of the grating and the mirror when the coherent laser is incident vertically onto the grating interferometric readout system. Since the mirror is parallel to the grating, the position of the laser output will not change. In addition, the light intensity can be carefully measured by using a photodiode in a fixed position. The appropriate detector location according to the maximum intensity of the first-order output laser beam was selected as follows:

(4) $$I_{1}=\frac{2I_{in}}{\pi^{2}}(1-\cos\frac{4\pi d}{\lambda })$$

where *d* is the directional displacement of the reflecting mirror, $I_{in}$ and λ are the intensity and wavelength of the incident, respectively.

Equation (4) shows that the period of every diffraction order intensity is λ/2. The optimal operating point can be obtained by adjusting the displacement *d*. At the optimal operating point, the system sensitivity reaches the maximum, and the response shows a linear trend. As for this optical displacement readout, the tiny displacement of the reflecting mirror with high sensitivity and accuracy can be easily detected. The proof mass covered by a high-reflective film serves as the reflecting mirror in our device.

The total noise of the accelerometer is composed of mechanical thermal noise and electrical noise. Generally, mechanical noise can be very low and the main noise of the accelerometer is limited by the electrical part. The electrical noise equivalent acceleration can be expressed as follows [30]:

(5) $$NEA_{electronics}=\frac{Noise_{electronics}}{G_{a-d}G_{d-I}}$$

where *G_d−I_* is the scale factor of the displacement transducer, and *G_a−d_* is the scale factor of the spring-mass system. The intensity changes can be detected by using a low-noise amplifier circuit and differential detection scheme [24,31], and *Noise_electronics_* can be reduced to a low level. According to Equation (4), the optical transducer will get an ultra-high scale factor of the displacement transducer that is obtained by the optical interference method. This can lead to a low electrical noise equivalent acceleration (*NEA_electronics_*), which is the dominant noise in most MEMS accelerometers [31,32,33,34,35,36].

#### 2.2.1. Micro Grating Interference Measurement System Design

The grating interference cavity is sensitive to the change in cavity length. In order to improve the measurement accuracy from the nm level to the pm level, the period of the micro-grating, the gap of the micro-cavity, the reflectivity of the grating, the selection of the reflecting film, and the material, etc., need to be optimized. The grating period and micro-cavity gap are related to the mechanism of the optical displacement sensor, and the selection of grating and film layer is related to the contrast of the emitted light intensity and the displacement sensitivity.

The parameters and materials of the cavity were selected and optimized by combining scalar theory with vector calculation software to improve the displacement sensitivity of the cavity and the contrast of the output light intensity. Microcavity schemes based on diffraction gratings can also improve accuracy by at least one order of magnitude to the sub-nanometer level through modulation and demodulation [17].

The output intensity of a subwavelength grating cavity is more sensitive to parameters. Combined with the specific structure of the microcavity, the period, gap, grating, reflective film material, and other parameters of the micro-grating were designed and optimized. The tools of parameter optimization include a finite-difference time-domain algorithm which strictly coupled wave theory and vector theory calculation.

#### 2.2.2. Simulation Analysis

**Figure 5:**
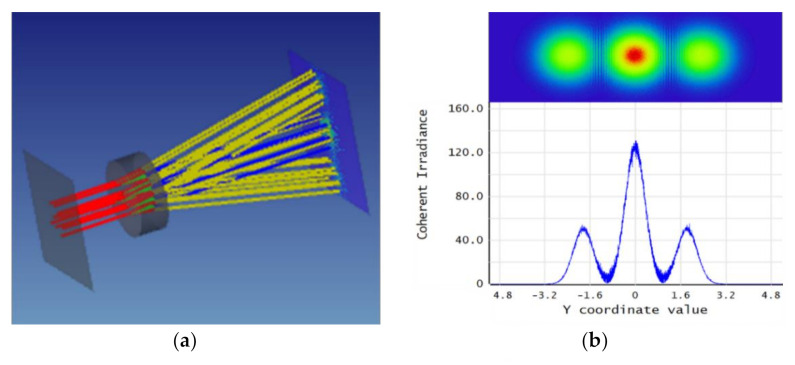
(**a**) Light path simulation with microcavity interference. (**b**)The simulation figure of micro light intensity diffraction pattern with a 0, +1, and −1 level. The *Y*-axis is along the direction in which the diffraction orders separate.

Scalar optics can accurately predict the propagation of light though linear, homogeneous, nondispersive media, but may fail to accurately capture the effects of the interaction of light with boundaries between different media. In such cases, taking a vector electromagnetics approach is more appropriate even when facing the cost of additional computations. ZEMAX software is used to analyze the grating structure and simulate the micro-optical cavity interference measurement system. ZEMAX’s hybrid non-sequence module combines scalar optics and vector electromagnetic waves for analysis. The transmittance and deflection of light rays at the grating interface are determined by the material properties and electromagnetic boundary effects, and the free space propagation is simulated using scalar theory. This simulation can accurately determine the position and distribution of interference light and reduce the time required for simulation. We designed the micro-grating with the form of 13 × 13 mm, including an aperture of 4 mm^2^. The material was a melting quartz, the surface was a chromium film for reflection, and the grating period was 4 μm. The simulation results are shown in Figure 5.

**Figure 6:**
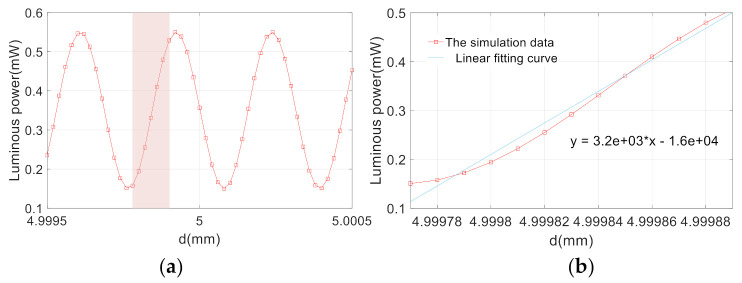
(**a**) Displacement-intensity curve for the +1 level. (**b**) Fitting of displacement-intensity magnification curve for the +1 level.

Then, the reflection mirror was scanning along the principal axis of acceleration, the +1 order spots were analyzed and the obtained curve of the displacement light intensity is shown in Figure 6a. The linear part of the curve was then amplified and fitted. The results obtained are shown in Figure 6b.

The formula of displacement and light intensity magnification that was obtained by fitting is as follows:

(6) $$I_{+1}=3226.7d-16133$$

In combination with subsequent signal processing system, the accuracy error of the light intensity detection is less than 1%. The sensitivity of the accelerometer can be expressed as follows:

(7) $$Sensitivity=\frac{\Delta V}{\Delta a}=\frac{\Delta I\times S_{p}\times G_{I-V}\times M}{\Delta d}\approx 60V/g$$

where Sensitivity=ΔV/Δa represents the sensitivity of unit acceleration converted to voltage value, $S_{p}$ = 0.54 A/W is the photosensitivity at working wavelength,$G_{I-V}$ = 32 V/A is the current-to-voltage conversion gain, M=Δd/Δa = 169 μm is the displacement-acceleration magnification and ΔI/Δa = 3226 W/m according to Equation (6). The system can obtain the acceleration-voltage amplification output sensitivity of about 60 V/g.

## 3. Experiments and Results

### 3.1. Device Processing

#### 3.1.1. MEMS Sensor Chip

MEMS devices require a low elasticity coefficient and a large mass, and high symmetry between the upper and lower springs of the devices has to be ensured in case of other incidents in the manufacturing process. In this work, to achieve this goal, we used the customized five-layer Silicon-On-Insulator (SOI) wafer as the starting point of the processing. The starting SOI was a (100) orientation, a 4-inch diameter wafer with two 2 μm buried oxide layers, and a 472 μm device layer sandwiched between two 12 μm substrate layers. Every three S-shaped cantilevers were fabricated on each substrate layer in a staggered distribution, and the proof mass was formed by the wafer thickness.

**Figure 7:**
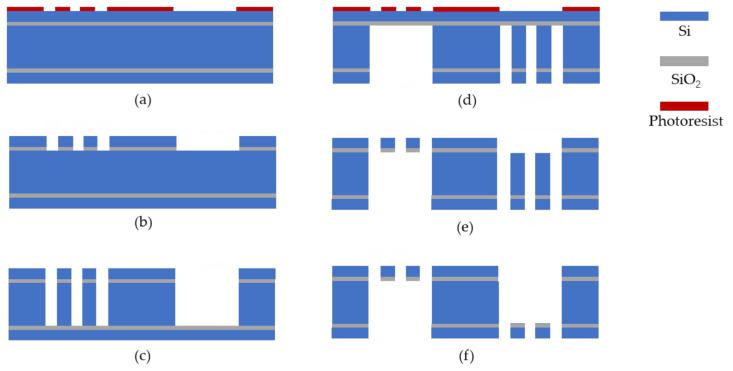
(**a**)–(**f**) Cross-sectional view of the fabrication process flow of the MEMS sensing chip.

According to Figure 7a, one side of the wafer was spun cast with a 10 μm thick photoresist (AZ4620) and lithographed. The developed substrate layer was then etched vertically onto the embedded oxide layer by an etching system to form the above pattern of three S-shaped cantilevers and substrate, with a thickness precisely limited to 12 μm. The exposed buried oxide layer was then removed with buffered HF as shown in Figure 7b. This silicon device layer was then etched to the next buried oxide layer by deep reactive ion etching (DRIE) to form a proven mass (Figure 7c). Another lithography was then performed using a bilateral alignment technique to pattern the lower three S-shaped cantilevers and the substrate on the back of another substrate layer, as shown in Figure 7d. The substrate layer was then etched onto the buried oxide layer and the exposed buried oxide layer was removed with buffered HF, as shown in Figure 7e. Subsequently, the substrate was also deeply etched by DRIE 472 μm to the buried oxide layer (Figure 7f). A final approach was to remove the remaining exposed buried oxide layer with buffered HF.

**Figure 8:**
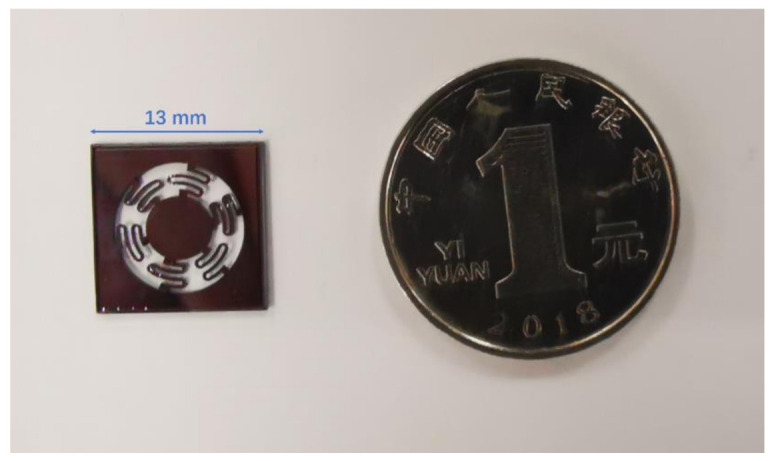
Photograph of the fabricated MEMS sensing chip.

According to Figure 8, the highly symmetrical micromachined sensing chip with an overall size of 13 mm × 13 mm × 500 μm was implemented after these processes. The sensing chip was composed of a proof mass with Φ (diameter) 5300 μm × 500 μm, and six S-shaped cantilevers with a 12 μm thickness symmetrically located around the proof mass. The MEMS mechanical sensing chip needs to possess high acceleration displacement sensitivity along the sensitive axis, low cross-axis sensitivity, a proper bandwidth, and a dynamic range. By adopting the fabrication processes, the cross-axis sensitivity could be greatly reduced, and the requirements for the alignment accuracy of the set were not high.

#### 3.1.2. Micro-Grating Chip

**Figure 9:**
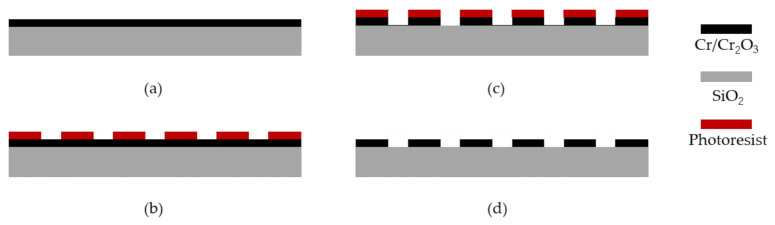
(**a**)–(**d**)Fabrication process steps used to prototype the two-region diffraction micro-grating chip.

The frame size of the two-region diffraction micro grating was 13 × 13 mm, which is consistent with the MEMS structure. The aperture was 4 mm^2^ and the grating period was 4 μm with a duty cycle of 2/3. The grating was produced by a quartz substrate with a periodic reflective film. After the parameters of the designed grating were determined, the layout design was carried out in consideration of the actual processing experience and equipment’s processing capacity. The grating mainly adopts the surface processing technology while the thickness and uniformity of each mask (photoresist and Cr/Cr2O_3_ film, etc.) need to be guaranteed. The error in each step of the process was minimized to ensure the precision of the grating in the preparation process. See Figure 9 for details.

The two-region stepped grating was prototyped on a double-side polished fused silica (SiO_2_) wafer using typical microfabrication techniques. A schematic illustration of the fabrication workflow used is shown in Figure 9. Firstly, the double-sided polished substrate glass substrate material was cleaned and dried, and a 130-nm-thick Cr/Cr_2_O_3_ layer was deposited as a mask. Photoresist spinning, baking, lithography, and the development process were then completed. The wet etching process was then carried out to remove the Cr/Cr_2_O_3_ layer after development. Finally, the residual photoresist was removed and the wafer was sawed into slices by a precision dicing machine.

**Figure 10:**
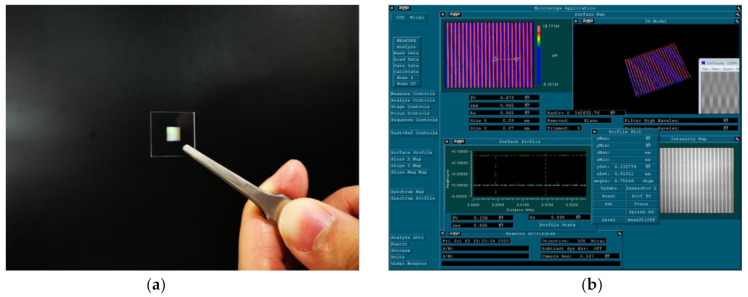
(**a**) Photograph of a fabricated sample. (**b**) Morphology depth characterization diagram of the grating using a ZYGO white light interferometer.

The microstructure of the grating was observed by a ZYGO white light interferometer, as shown in Figure 10. The actual depth after fabrication was 132.794 nm, which was consistent with the designed depth.

### 3.2. System Testing

**Figure 11:**
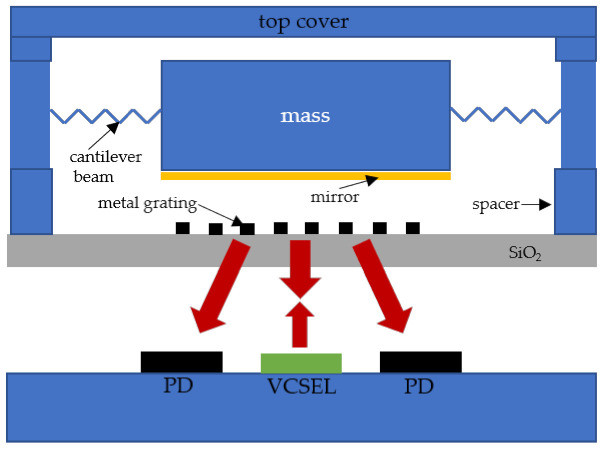
Schematic diagram of the internal structure of the sensor.

The MEMS sensitive chip, grating chip, VCSEL laser, and Si photodiodes through the package structure were integrated, and the laser drive and signal readout circuit kept completing the integration of the whole prototype accelerometer, as shown in Figure 11. A VCSEL (850 nm wavelength, Model-L850VG1, Thorlabs Newton, NJ, USA) was collimated by an aspherical lens and irradiated the grating of the sensing probe. The 0 and ±1 order diffraction intensities were detected by several Si photodiodes(s10625-01ct, Hamamatsu). The high stability control of VCSEL were realized by the circuit with power fluctuations about 1.2‰ over 24 h, and the photocurrent subtraction method was used to suppress the noise of the laser relative intensity noise (RIN) [6,37].

**Figure 12:**
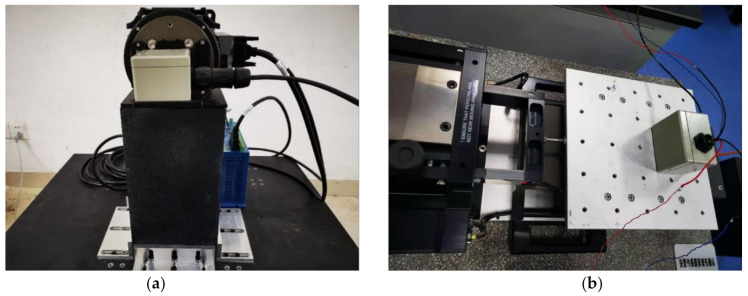
(**a**) Static sensitivity measurement using a high precision turntable ARMS150. (**b**) Dynamic sensitivity measurement using a low frequency vibrator APS129.

A high precision turntable ARMS150, a low frequency vibrator APS129, and the dynamic signal analyzer 3560-D were then used to test the sensor’s response sensitivity, which is shown in Figure 12. The test results were similar to the design simulation result, with an error of about 10%. The error may have been caused by the actual interference beam, which may not match the setup in the simulation perfectly. Meanwhile, the sensitivity amplitude decreases slightly as the frequency approached the resonant frequency of the device, as shown in Table 3.

**Figure 13:**
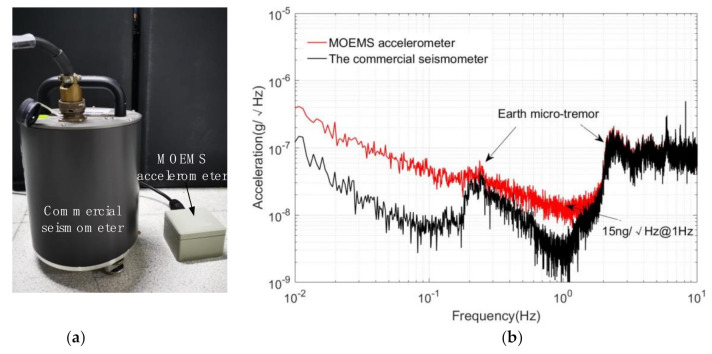
(**a**) Calibrating the self-noise of the MEMS accelerometer with a commercial seismometer for reference. (**b**) The power spectral density (PSD) of the output of the MOEMS accelerometers and the commercial seismometer.

The MOEMS accelerometer and the commercial seismometer (CMG-3EPS), as the reference instrument [38], were simultaneously placed on the vibration isolation foundation of the laboratory for long-term data acquisition with a sampling rate of 200 Hz, as shown in Figure 13a. The noise spectral density curves of the MOEMS accelerometer and commercial microseismometer are shown in Figure 13b. In the figure, the red line is the MOEMS accelerometer, and the black line is the commercial high-precision seismometer. Both of them picked up the ground pulsation signal (0.2~0.3 Hz, 2~3 Hz characteristic peak) in the laboratory area at the same time. The MOEMS accelerometer developed in the experiment obtained the ultra-low self-noise of 15 ± 0.1 ng/√Hz @ 1 Hz, as shown in Figure 13b. This accelerometer is one of the most sensitive MOEMS accelerometers in the world, as shown in Table 4.

## 4. Conclusions

This paper proposes an optimal mechanical design to implement an MOEMS accelerometer based on a grating interferometry cavity and a micromachined sensing chip. Due to the acceleration-sensitive MEMS sensor chip, the compact grating interferometer, and the modified processing circuit, the MOEMS accelerometer can achieve high sensitivity and resolution in acceleration measurements. In order to achieve ultra-high acceleration sensitivity in the accelerometer, the structure was redesigned and optimized based on analytical and FEM approaches. The experimental results indicate that this MOEMS accelerometer with the proposed design can achieve an a–d sensitivity of about 169 μm/g and an acceleration sensitivity of about 60 V/g. The actual test results show that the self-noise of this MOEMS accelerometer is below 15 ng/√Hz @1 Hz. These levels present a significant improvement over previous works. This optical accelerometer is expected to be used in seismic surveys and other fields in the future by applying the developed models and discovering high performance compact designs with streamlined packaging procedures.

## Figures and Tables

**Table 1:** Physical dimensions of the Micro-Electro-Mechanical Systems (MEMS) sensor chip.

Symbol	Parameter	Value	Symbol	Parameter	Value
w	cantilever beam width	300 µm	$R_{m}$	Mass radius	2650 µm
*t*	cantilever beam thickness	12 µm	*T*	Mass thickness	500 µm
$Deg_{s1}$	supporting beam angle 1	15°	$r_{n}$	Inner radius of the nth cantilever	$R_{m}+(n-1)\times w$
$Deg_{s2}$	supporting beam angle 2	7°	$R_{n}$	Outer radius of the nth cantilever	$R_{m}+n\times w$
$Deg_{c1}$	cantilever beam angle 1	14°	*L*	Frame length	13,000 µm
$Deg_{c2}$	cantilever beam angle 2	25°	*W*	Frame width	13,000 µm

**Table 2:** Ratio of insensitive axis frequency to sensitive axis frequency based on the micro-mechanical sensor structure. (A decimal approximation).

$f_{z}(\mathrm{Hz})$	$f_{x}/f_{z}$	$f_{y}/f_{z}$	$f_{\alpha }/f_{z}$	$f_{\beta }/f_{z}$	$f_{\gamma }/f_{z}$
24.8	13.0	13.0	23.6	23.6	23.6

**Table 3:** Sensitivity amplitude test results. (Two significant digits are taken for the results of the experiment).

Frequency/Hz	DC	0.1	0.5	1	5	10
Sensitivity amplitude/(V/g)	54.42	54.39	53.32	51.24	42.28	40.60

**Table 4:** The self-noise comparison between the proposed MOEMS accelerometer and other MOEMS accelerometers.

Accelerometers	California Institute of Technology [7]	Sandia National Laboratories [15]	University of Glasgow [3]	Beihang University [22]	This Work
Specification	Photonic-crystal nanocavity	Optomechanical	Light-intensity	Grating diffraction	Grating diffraction
Self-noise (ng/√Hz)	10^4^	43.7 (thermal noise floor)	41	137	15

## Data Availability

The data presented in this study are available on request from the corresponding author.
